# Improper modification and cleavage leads to irregular aggregation of TDP‐43 in Neurons

**DOI:** 10.1002/alz.092582

**Published:** 2025-01-09

**Authors:** Joyal Xavier, Meenakshi Singh, Sampada P Tamhankar, Raj Amin

**Affiliations:** ^1^ Auburn University, Auburn, AL USA

## Abstract

**Background:**

TAR‐DNA‐binding protein 43 (TDP43), is a pathologic marker in neurodegenerative diseases including frontotemporal lobar degeneration and amyotrophic lateral sclerosis. The aggregation of TDP‐43, a crucial RNA‐binding protein, is a consequence of post‐translational modifications (PTMs) that disrupt its normal function. PTMs such as phosphorylation and ubiquitination contribute to the aberrant accumulation of TDP‐43 aggregates, leading to neurodegenerative disorders like amyotrophic lateral sclerosis (ALS) and frontotemporal lobar degeneration (FTLD). This pathological process disrupts RNA metabolism and cellular homeostasis. The current study focuses on better understanding the mechanisms associated with TDP‐43 PTMs and aggregation. Findings from these studies are essential for developing targeted therapeutic strategies to mitigate neurodegenerative diseases associated with TDP‐43 pathology.

**Method:**

Overexpressing TDP‐43 in Ht22 hippocampal cells was accomplished by Lentiviral technology. TDP‐43 GFP over‐expressing stable cell lines were exposed to either an acidic environment (pH 6) or a neutral environment (pH 7.4) or sodium arsenate. Mitochondria, nuclei, and cytosolic fractions were isolated, and proteins were resolved by Western analysis to measure levels of TDP43. Asparaginyl endopeptidase (AEP) inhibitor (Compound 11), was used to determine its effect on aggregation of TDP43. The Aggregation assay kit was used to measure the levels of TDP‐43 aggregation. Physiological assays were used to measure the consequence of TD43 aggregation in the mitochondria (Seahorse, mitochondrial respiration) and nuclei (transcriptome analysis).

**Result:**

We have observed the localization of AEP in the mitochondria under normal physiological pH conditions as well as total and cleaved TDP43. However, under acidic conditions and following sodium arsenate exposure, we observed an increase in pTDP43 levels as well as full‐length TDP43 in the mitochondria and nucleus when compared to normal media. Alternatively, we observed a decrease in pTDP43 in the mitochondria and nucleus following compound 11 treatment in the acidic media or sodium arsenate.

**Conclusion:**

We observed an increase in pTDP43 in cells exposed to an acidic environment. We believe this leads to aggregation levels and helps explain the consequences of cleaved TDP43 on cellular energy regulation and cell viability. Consequently, compound 11 reduces the levels of TDP43 aggregation patterns in the mitochondria and nuclei.

